# The prognostic value of serum uric acid in the acute phase of hemorrhagic stroke patients in black Africans

**DOI:** 10.11604/pamj.2019.32.165.15107

**Published:** 2019-04-09

**Authors:** Yacouba Njankouo Mapoure, Chia Mark Ayeah, Hamadou Ba, Hugo Bertrand Mbatchou Ngahane, Romuald Hentchoya, Henry Namme Luma

**Affiliations:** 1 Department of Clinical Sciences, University of Douala, Douala, Cameroon; 2 Department of Internal Medicine, Douala General Hospital, Douala, Cameroon; 3 Department of Internal Medicine, Mboppi Baptist Hospital, Douala, Cameroon; 4 Department of Internal Medicine, University of Yaoundé I, Douala, Cameroon; 5 Service of Intensive Care Unit, Douala General Hospital, Douala, Cameroon

**Keywords:** Hyperuricemia, acute hemorrhagic stroke, mortality, functional outcome

## Abstract

**Introduction:**

Very few studies have been conducted to evaluate the prevalence of hyperuricemia and its impact on the prognosis amongst acute hemorrhagic stroke (AHS) patients. The objectives was to determine the prevalence of hyperuricemia in AHS patients and examined the association between hyperuricemia and stroke outcomes in the Douala General Hospital (DGH).

**Methods:**

This was a hospital based prospective cohort which included AHS patients with baseline SUA levels and 3 months post stroke follow-up data. SUA values were divided into quintiles. Associations between hyperuricemia and stroke outcomes were analyzed using multiple logistic regression and survival analysis (cox regression and Kaplan Meier).

**Results:**

A total of 221 AHS patients were reviewed with a mean age of 55.8±11.8 years. The prevalence of hyperuricemia among AHS patients was 34.4% with mean SUA level of 376.8±131.9 μmol/l. On multivariate analysis, hyperuricemia was not independently associated with early death [(OR = 1.072 (CI: 0.370-3.056; p = 0.897)] and poor functional outcome [(OR=2.487 (CI: 0.771-8.699; p = 0.154)] after hemorrhagic stroke. No significant increase in stroke deaths was observed across higher SUA quintiles amongst hemorrhagic stroke patients (p = 0.326). No statistically significant correlation was observed between SUA level and NIHSS (r = 0.063, p = 0.353) and between SUA level and mRS (r = 0.030, p = 0.662) in hemorrhagic stroke.

**Conclusion:**

About one third of patients present with hyperuricemia in the acute phase of hemorrhagic stroke. Hyperuricemia can act as risk factor for stroke because of its relationship with CVRFs but hyperuricemia has no impact on the severity and short-term outcome amongst black African hemorrhagic stroke patients.

## Introduction

Stroke is the second leading cause of death and the leading cause of adult disability worldwide [1, 2]. Stroke ranks 6^th^ out of the top ten causes of death and accounts for one of the top frequent neurological disease consulting at the neurology units [3]. The burden of stroke seems to be shifting to the developing world and currently two-thirds of stroke mortality cases occur in sub-Saharan Africa [4, 5]. In Africa, stroke case fatality ranges from 31.9% to as high as 69.7% when due to hemorrhagic stroke [6-8]. Uric acid is a powerful anti-oxidant that exerts neuroprotective effects by acting as a free radical scavenger in plasma [9-11]. It has been reported that increased levels of uric acid are associated with established cardiovascular risk factor such as elevated serum triglyceride and cholesterol concentration, hypertension, obesity, insulin resistance and metabolic syndrome [9, 12]. The role of SUA in acute stroke is poorly understood since some studies demonstrate that SUA is associated with adverse outcomes and mortality [13-18] while other studies are suggesting that SUA may be beneficial and protect against poor outcomes [19, 20]. Despite these controversies as to whether there is a relation between SUA levels and outcome after acute stroke, the role of SUA in the acute phase of hemorrhagic stroke only has received scant attention in world literature. Therefore, in this study we estimated the prevalence of hyperuricemia in hemorrhagic stroke and determined its relationship with CVRFs and stroke outcome within 3 months post stroke onset.

## Methods

**Patients and study design:** We carried out a hospital based prospective cohort study in a tertiary care hospital in Douala, Cameroon. We included consenting patients admitted for hemorrhagic stroke in the neurology unit of the department of internal medicine and the intensive care unit of the Douala General Hospital (DGH) from January 2010 to January 2016. This study was approved by the Institutional Ethics Committee of Research on Human Health of the University of Douala and the study hospital-DGH. Patients who were admitted for confirmed hemorrhagic stroke within 7 days of onset of symptoms were included in our study. Patients with incomplete files, acute ischemic stroke and cerebral venous thrombosis were excluded.

**Data collection and patient management:** Demographic data, including age, sex and relevant medical history such as hypertension (HTN), diabetes mellitus (DM), smoking history, alcohol abuse, use of diuretics, history of diseases like chronic kidney disease (CKD), gout and other cardiovascular events such as atrial fibrillation, congestive heart failure (CHF), coronary artery disease (CAD) and ischemic heart disease (IHD) were recorded. Baseline vital and anthropometric parameters such as blood pressure, pulse, respiratory rate, oxygen saturation weight, height and an abdominal circumference values were recorded using standard operating procedures. The following definitions and standard operating techniques were used to identify risk factors for stroke in each subject: hypertension was defined as patient with medical history of hypertension, treated or not and patient with persistent high blood pressure > 140/90 mmHg after stroke. Diabetes Mellitus was defined as patient with medical history of diabetes, treated or not, random serum glucose ≥ 11.1 mmol/l or venous fasting glucose test ≥ 6.99 mmol/l. Dyslipidemia was defined as patient with medical history of dyslipidemia or Total cholesterol (TC) > 5.2 mmol/l or Low-density lipoprotein (LDLc) > 2.59 mmol/l or High-density lipoprotein (HDLc) < 1.0 mmol/l in males or < 1.3 mmol/l in females, triglycerides (TG) > 1.7 mmol/l. The presence of the metabolic syndrome was determined using the new definition [21]. Alcohol abuse defined as daily alcohol intake > 40 g/l. Obesity was defined as patient with a body mass index (BMI) > 30 and when it's impossible to have the BMI, we use the abdominal circumference: >102 cm in male and >88 cm in female. Smoking was defined as patient with history of smoking (current smokers and ex-smokers) quantified in pack years. Sleep apnea syndrome (SAS) was defined as patient with a medical history of SAS diagnosed before or after stroke onset using pulse oximetry in patients with suggestive symptoms such as snoring, witnessed apnea, daytime sleepiness and the presence of comorbidities hypertension, heart failure and obesity. Patients with severe conditions like a Glasgow Coma Scale < 8/15 or septic shock were directly admitted in the ICU while other cases were hospitalized in the NU.

Blood sample was collected from all patients during the first 24 hours of admission to check SUA levels, fasting blood sugar, complete metabolic panel (urea, creatinine, uric acid, electrolytes etc) and lipid profile using the Cobas 311 autoanalyzers. A full blood count with platelet counts, prothrombin time, cephaline-kaolin time, C-reactive protein (CRP), erythrocyte sedimentation rate (ESR), and HIV serology were done. Other tests were prescribed if required by the patient's conditions: chest X-ray, urine culture, blood culture, and thick blood film to check for plasmodium falciparum. Neurological assessment was done by a neurologist or intensive care specialist. Interpretation of CT scans was done by both radiologist and neurologist and its findings were recorded. The type of stroke was also recorded. Electrocardiography was systematically done for hypertensive patients with hemorrhagic stroke. On admission and at 3 months post stroke, the Glasgow coma scale (GCS) and the National Institute of Health Stroke Scale (NIHSS) were used to determine the stroke severity while the functional outcome was evaluated by the modified Rankin score (mRS). Stroke death and stroke recurrence during admission and within 3 months post stroke was also recorded. In case of death, the staff had to be precise about the cause of death. Poor (bad) functional outcome was considered in patients with mRS > 2 within the first 3 months post stoke discharge while good functional outcome was considered in patients who are alive within the first 3 months post stroke and with mRS ≤ 2. Follow up was done daily for clinical evaluation and complications were noted. Oxygen was administrated if ambient oxygen saturation was less than 94%. Paracetamol was used 1g every six hours if temperatures higher than 37.5^0^C were noted. Prevention of deep venous thrombosis and stress ulcers was done using prophylactic dose of enoxaparin (40mg) and omeprazole (20mg), respectively. An insulin protocol was set up when capillary glycaemia was above 7.8 mmol/l. Concerning the blood pressure, nicardipine was given intravenously with electric syringe in case of high blood pressure with a target of 140 to 160mmHg for systolic blood pressure in hemorrhagic stroke. Antibiotics and artemether were used for bacterial infection and malaria, respectively.

**Statistical analysis:** Statistical analysis was done using the Statistical Package for Social Sciences (SPSS) Standard version, Release 20.0 (IBM Inc. 2012). Mean and standard deviation (SD) of all continuous data are reported. SUA was a scale variable but was categorized into two groups, those having normal and high SUA levels (hyperuricemia) as follows; normal SUA range was SUA ≤ 356.9 μmol/l in females and SUA ≤ 416.4 μmol/l in males while high SUA range was SUA > 356.9 μmol/l in females and SUA > 416.4 μmol/l in males. SUA was also divided into quintiles (mean SUA level per quintile range) as follows: Q1 ≤ 291.5 μmol/l (249.4 μmol/l), Q2 = 297.4-356.9 μmol/l (329.6 μmol/l), Q3 = 362.8-416.4 μmol/l (391.6 μmol/l), Q4 = 422.3-499.7 μmol/l (457.5 μmol/l) and Q5 ≥ 505.6 μmol/l (627.3 μmol/l). Hyperglycemia was defined as glucose levels > 7.8 mmol/l), high TC considered as TC levels > 5.2 mmol/l, high LDLc considered as LDLc levels > 2.59 mmol/l or decreased HDLc considered as HDLc levels < 1.0 mmol/l in males or < 1.3 mmol/l in females and high TG considered as TG levels > 1.7 mmol/l. Independent Samples t-test was used to assess differences in continuous variables since the normality assumption was not violated following Kolmogorov-Smirnov and Shapiro-Wilk test for normality. Cramer's V, Chi Square test and Fisher's Exact Test were used for categorical variables. Univariate analysis was first performed with demographic characteristics and the risk factors of stroke by cross-tabulations with X^2^ or Fisher's exact tests for the unadjusted odds ratios (ORs) and then multiple logistic regression was done to adjust the confounding effects of the dependent predictors of death during admission. All predictor variables with p values < 0.2 that were gotten from the univariate analysis were included in our multivariate analysis. Survival analysis was performed using Kaplan Meier and Cox regression analysis. Level of significance was considered 0.05 (two-sided).

## Results

**Basic characteristics of the study population:** Table 1 shows the basic characteristics of the study population: A total of 221 patients with hemorrhagic stroke were included amongst which 134 were males (60.6%). The mean age was 55.8±11.8 years with most patients aged 46-60 years (49.8%). Table 1 lists the demographics, the vascular risk factors and clinical parameters of the study population. The most prevalent vascular risk factors were hypertension and hyperlipidemia, which were present in 96.8% and 66.5% of the patients respectively. About 14.0% of the study population had experienced another stroke in the past. The prevalence of hyperuricemia in hemorrhagic stroke patients was 34.4% (76/221). Of the 221 participants, the prevalence of CKD was seen in 2.7%. The mean SUA level was 376.5±131.8 μmol/l, while the mean SUA levels in the male patients (392.8±121.1 μmol/l) was significantly higher than amongst the female patients (352.1±144.1 μmol/l) with p=0.024. The proportion of females with high SUA level [33(37.9%)] was lower than the proportion of females with normal SUA level [54(62.1%)]. Among the males, the trend was the same, with a lower proportion of 43 (32.1%) males having high SUA levels as compared to 91 (67.9%) with normal SUA levels.

**Table 1:** Basic characteristics of the study population

Variables	Values
Male, n (%)	134 (60.6)
Age, years (Mean±SD)	55.8±11.8
Hypertension, n (%)	214 (96.8)
Diabetes Mellitus, n (%)	53 (24.0)
Smoking, n (%)	36 (16.3)
Alcohol Intake, n (%)	85 (38.5)
Dyslipidemia, n (%)	147 (66.5)
Obesity, n (%)	48 (21.7)
Metabolic Syndrome, n (%)	43 (19.5)
Migraine, n (%)	14 (6.3)
Sleep Apnea Syndrome, n (%)	19 (8.6)
Use of Oral Contraceptives, n (%)	1 (0.5)
Coronary Artery Disease, n (%)	7 (3.2)
Atrial fibrillation, n (%)	7 (3.2)
Heart disease, n (%)	12 (5.4)
Valvulopathy, n (%)	5 (2.3)
Past Stroke, n (%)	31 (14.0)
Sedentary Lifestyle, n (%)	18 (8.1)
Family History of stroke, n (%)	8 (3.6)
Hyperuricemia, n (%)	76 (34.4)
Chronic kidney disease, n (%)	6 (2.7)
HIV Seropositive, n (%)	6 (2.7)
Systolic Blood Pressure (Mean±SD), mmHg	178.9±34.4
Diastolic Blood Pressure (Mean±SD), mmHg	107.6±22.1
Body Mass Index (Mean±SD), Kg/m^2^	30.3±5.6
Glasgow Coma score (Mean±SD)	11.4±3.8
NIHSS Score (Mean±SD)	16.5±10.1
Modified Rankin Score (Mean±SD)	3.7±1.3
Serum Uric Acid (Mean±SD), μmol/l	376.5±131.8
Serum Uric Acid in males (Mean±SD), μmol/l	392.8±121.1
Serum Uric Acid in females (Mean±SD), μmol/l	352.1±144.1
Creatinine (Mean±SD), μmol/l	160.9±38.4
Urea (Mean±SD), mmol/l	2.4±0.7
Glycemia (Mean±SD), mmol/l	7.9±4.0
Glycated hemoglobin (Mean±SD), %	6.8±1.7
Total Cholesterol (Mean±SD). mmol/l	5.1±0.7
Triglycerides (Mean±SD). mmol/l	1.3±0.6
HDL Cholesterol (Mean±SD). mmol/l	1.3±0.5
LDL Cholesterol (Mean±SD). mmol/l	3.3±1.6

NIHSS: National Institute of Health Stroke Scale, HDL: High density lipoprotein cholesterol, LDL: Low density lipoprotein cholesterol, SD: Standard deviation

**Figure 1:**
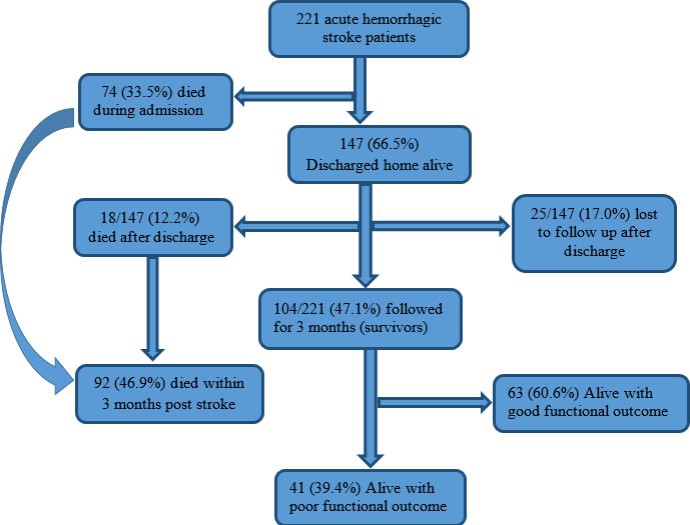
Flow chart on functional outcome and death during three-month follow up

**Outcome in the acute phase and 90 days post stroke:** The outcome of patients within the 90 days post stroke has been assessed in Figure 1. In this series, 74 out of 221 patients (33.5%) died during admission and 147 patients (66.5%) discharged home alive were eligible for follow up amongst which 12.2% (18/147) died within three months and 17.0% (25/147) were lost to follow up. Among stroke survivors (n=104), 60.6% and 39.4% had good and bad functional outcomes. Overall, the 3 months post stroke mortality was 46.9% (92/196). The mean SUA concentration ± SD (standard deviation) of stroke survivors of 354.9 ± 91.7 μmol/l was significantly lower than that of those who died 388.8 ± 118.0 μmol/l, p-value=0.025. The mean ± SD duration of hospital stay and of follow up was 9.7 ± 7.0 days and 49.9 ± 40.7 days respectively.

**Correlation between SUA and clinical data on admission:** Table 2 shows the correlation between SUA and clinical data on admission; there was a weak negative significant correlation between age and SUA concentration (r = -0.158*, p=0.019, n=221) and there was also a weak negative significant correlation between Glasgow coma score and SUA concentration (r=-0.137*p = 0.042, n = 221). SUA concentrations had strong positive significant correlations with systolic blood pressure (r = 0.218**p = 0.001, n = 221), diastolic blood pressure (r = 0.236**, p =< 0.001, n = 221), creatinine (r = 0.532**, p =< 0.001, n = 221), urea (r = 0.234**, p =< 0.001, n = 221), triglycerides (r = 0.188**, p = 0.009, n = 191) and low density lipoprotein cholesterol (r = 0.165*, p = 0.022, n = 191). There was a positive but insignificant correlation between glycemia levels and SUA concentration (r = 0.114, p = 0.091, n = 221). There was no significant correlation between length of hospital stay and SUA concentration (r = 0.093, p = 0.167, n = 221).

**Table 2:** Correlation between SUA and clinical data on admission

Variables	R	P value	Total
Age	-0.158*	0.019	221
Systolic blood pressure	0.218**	0.001	221
Diastolic blood pressure	0.236**	<0.001	221
Body Mass Index	0.164	0.171	71
NIHSS	0.063	0.353	221
mRS	0.030	0.662	221
Glasgow coma score	-0.137*	0.042	221
Creatinine	0.532**	<0.001	221
Urea	0.234**	<0.001	221
Glycemia	0.114	0.091	221
Total cholesterol	-0.095	0.191	191
Triglycerides	0.188**	0.009	191
High density lipoprotein cholesterol	0.077	0.290	191
Low density lipoprotein cholesterol	0.165*	0.022	191
Length of hospital	0.093	0.167	221
Duration of follow up	-0.163*	0.015	221

NIHSS: National Institute of Health Stroke Scale, mRS: Modified Rankins Scale, R: Correlation coefficient

**Comparison of SUA levels with CVRFs amongst hemorrhagic stroke patients:** Table 3 shows the comparison of SUA levels with CVRFs amongst hemorrhagic stroke patients: Significant associations were observed between SUA levels and the following CVRFs; alcohol abuse [p = 0.049], dyslipidemia [p = 0.011], obesity [p = 0.026], metabolic syndrome [p = 0.026] and chronic kidney disease [p = 0.019].

**Table 3:** Comparison of serum uric acid levels with CVRFs in stroke patients

Variable	Normal SUA	High SUA	Total	aOR (95% CI)	P value
Age > 45 years	119 (82.1)	60 (78.9)	179	0.819 (0.409-1.643)	0.574
Male gender	91 (62.8)	43 (56.6)	134	0.773 (0.440-1.360)	0.372
Hypertension	138 (95.2)	76 (100.0)	214	Undefined	0.099*
Diabetes Mellitus	29 (20.0)	24 (31.6)	53	1.846 (0.980-3.474)	0.055
Smoking	22 (15.2)	14 (18.4)	36	1.263 (0.605-2.636)	0.534
Alcohol abuse	49 (57.6)	36 (47.4)	85	1.763 (1.001-3.108)	0.049
Dyslipidemia	88 (60.7)	59 (77.6)	147	2.248 (1.192-4.238)	0.011
Obesity/Overweight	25 (17.2)	23 (30.3)	48	2.083 (1.085-3.998)	0.026
Metabolic syndrome	22 (15.2)	21 (27.6)	43	2.137 (1.085-4.202)	0.026
Diuretic use	26 (18.4)	17 (23.0)	43	1.319 (0.662-2.627)	0.430
Coronary artery disease	6 (4.1)	1 (1.3)	7	0.309 (0.163-2.365)	0.426*
Atrial fibrillation	4 (2.8)	3 (3.9)	7	2.851 (0.541-15.014)	0.694*
Heart disease	9 (6.2)	3 (3.9)	12	1.586 (0.488-4.342)	0.552*
Valvulopathy	4 (2.8)	1 (1.3)	5	0.470 (0.052-4.281)	0.662*
Previous stroke	20 (13.8)	11 (14.5)	31	1.058 (0.478-2.341)	0.890
Chronic Kidney Disease	1 (0.7)	5 (6.6)	6	10.141 (1.163-88.445)	0.019*

aOR: unadjusted odds ratio, CI: confidence interval

**Figure 2:**
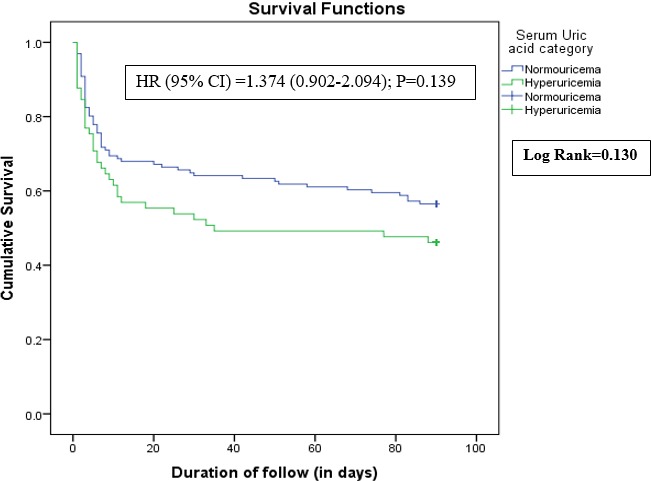
Graph showing a Kaplan-Meier survival rate of patients with normouricemia compared with hyperuricemia after hemorrhagic stroke

**Figure 3:**
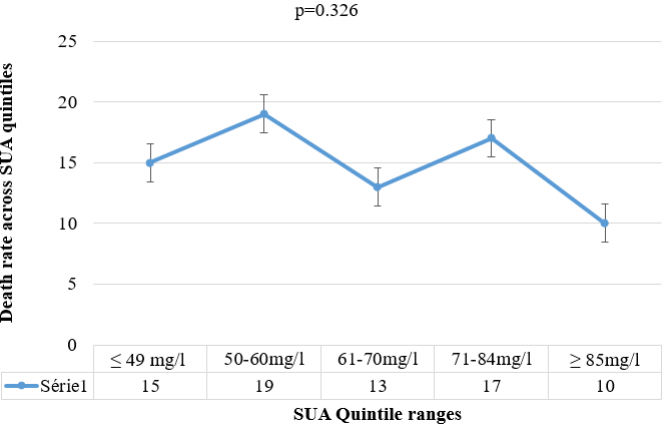
Shows the death rate during admission across SUA quintiles

**Predictive value of SUA on mortality amongst hemorrhagic stroke patients:** On univariate analysis, the following were factors significantly associated with death in acute hemorrhagic stroke: lack of insurance, history of smoking, alcohol abuse, dyslipidemia, metabolic syndrome, hyperglycemia, low HDLc, increased triglycerides, high total cholesterol, GCS less than 9, NIHSS > 14, mRS > 2 and presence of in-hospital complications (p < 0.05). On univariate analysis, there was no significant association between high SUA levels and death among acute hemorrhagic stroke patients [(p = 0.172, OR = 1.515, 95% CI: 0.833-2.753)]. On multivariate analysis, no such independent association was seen between hyperuricemia and mortality amongst hemorrhagic stroke patients [(OR = 1.072 (CI: 0.370-3.056; p = 0.897)]. After multivariate analysis, only GCS less than 9, NIHSS greater than 14 and hyperglycemia remained as significant independent predictors of death within 3 months amongst hemorrhagic stroke patients (p < 0.05). A history of HTN was a protective factor against death amongst hemorrhagic stroke patients. Furthermore, no independent association between increasing SUA concentration and survival was noted with the Kaplan Meier curve with adjusted HR (95% CI) of 1.374 (0.902-2.094); P = 0.139, as shown in Figure 2. As shown in Figure 3, the proportion of the death on admission amongst hemorrhagic stroke patients did not significantly increase with high levels of SUA (P = 0.326). The proportion of deaths in the 2^nd^ SUA quintile range (SUA = 297.4-356.9 μmol/l) and 4^th^ quintile range (SUA = 422.3-499.7 μmol/l) had the highest proportion of deaths, 19 (25.7%) and 17 (23.0%) respectively while the 5^th^SUA quintile range had the lowest proportion of deaths on admission, 10 (13.5%) (Table 4).

**Table 4:** Independent predictors of death within 3 months post stroke

Variable	Functional outcome		Univariate analysis		Multivariate analysis	
	Survivor	Death	Unadjusted OR (95% CI)	P Value	Adjusted OR (95% CI)	P Value
AGE > 45 years	82 (78.8)	77 (83.7)	1.377 (0.666-2.847)	0.387	--	--
Male gender	67(64.4)	52(56.4)	0.718(0.404-1.276)	0.258	--	--
Unemployed	62(59.6)	44(47.8)	0.621(0.353-1.094)	0.098	0.788 (0.271-2.294)	0.662
Unmarried	17(16.3)	17(18.5)	1.160(0.554-2.431)	0.694		
Insurance	33(31.7)	16(17.4)	0.453(0.230-0.893)	0.021	0.804 (0.245-2.638)	0.719
Hypertension	103(99.0)	88(95.7)	0.214(0.023-1.947)	0.189	0.063 (0.005-0.863)	0.038
Diabetes Mellitus	21(20.2)	28(30.4)	1.729(0.900-3.322)	0.098	1.114 (0.333-3.724)	0.861
Smoking	24(23.1)	9(9.8)	0.361(0.158-0.825)	0.013	1.817 (0.465-7.092)	0.390
Alcohol abuse	53(51.0)	27(29.3)	0.400(0.221-0.772)	0.002	0.414 (0.141-1.220)	0.110
Dyslipidemia	79(76.0)	50(54.3)	0.377(0.205-0.693)	0.001	0.402 (0.126-1.284)	0.124
OBESITY	30(28.8)	12(13.0)	0.377(0.205-0.693)	0.822	--	--
Metabolic syndrome	28(26.9)	9(9.8)	0.294(0.131-0.664)	0.002	0.702 (0.032-15.632)	0.823
CAD/IHD	4(3.8)	2(2.2)	0.556(0.099-3.106)	0.686	--	--
Atrial fibrillation	2(1.9)	4(4.3)	2.318(0.415-12.962)	0.422	--	--
Heart disease	6(5.8)	5(5.4)	0.939(0.277-3.184)	0.919	--	--
Valvulopathy	3(2.9)	0(0.0)	Undefined	0.249	--	--
Recurrent stroke	16(15.4)	10(10.9)	0.671(0.288-1.562)	0.352	--	--
CKD	1(1.0)	5(5.4)	5.920(0.679-51.636)	0.101	2.377 (0.150-37.742)	0.539
GCS<9	6(5.8)	56(60.9)	25.407(10.080-64.045)	<0.001	6.930 (2.199-21.836)	0.001
Hyperurcemia	30(28.8)	35(38.0)	1.515(0.833-2.753)	0.172	1.072 (0.376-3.056)	0.897
Complications	37(35.6)	57(62.0)	2.949(1.648-5.276)	<0.001	1.792 (0.713-4.502)	0.214
NIHSS>14	31(229.8)	80(87.0)	15.699(7.505-32.840)	<0.001	4.166 (1.432-12.121)	0.009
mRS>2	69(66.3)	90(97.8)	22.826(5.306-98.198)	<0.001	5.351 (0.789-36.274)	0.086
Hyperglycemia	24(23.1)	41(44.6)	2.680(1.450-4.952)	0.001	4.178 (1.356-12.872)	0.013
Elevated Urea	17(16.3)	33(35.9)	2.862(1.462-5.605)	0.002	1.237 (0.322-4.752)	0.757
Elevated Creatinine	26(25.0)	34(37.0)	1.759(0.952-3.248)	0.070	1.412 (0.351-5.681)	0.627
High LDLc	76(73.1)	71(77.2)	1.246(0.649-2….390)	0.509	--	--
High TC	46(44.2)	56(60.9	1.961(1.109-3.469)	0.020	1.206 (0.420-3.461)	0.728
High TG	14(13.5)	38(41.3)	4.524(2.248-9.105)	<0.001	2.184 (0.705-6.765)	0.176
Decreased HDLc	61(58.7)	32(34.8)	0.376(0.211-0.672)	0.001	0.559 (0.210-1.487)	0.244

CAD/IHD; coronary artery/ischemic heart disease, CKD; chronic kidney disease, GCS; Glasgow coma score, NIHSS: National Institute of Health Stroke Scale, mRS: modified Rankin scale, HDL: High density lipoprotein cholesterol, LDL: Low density lipoprotein cholesterol

**Predictive value of SUA on functional outcome in hemorrhagic stroke:** On univariate analysis, the following were factors significantly associated with poor functional outcome in hemorrhagic stroke: history of heart disease, GCS less than 9, NIHSS >14 and mRS > 2 (p < 0.05). On univariate analysis, there was no significant association between high SUA levels and with poor functional outcome amongst acute hemorrhagic stroke patients [(p = 0.187, OR=1.846, 95% CI: 0.781-4.364)]. On multivariate analysis, no such independent association was seen between hyperuricemia and with poor functional outcome amongst hemorrhagic stroke patients [(OR = 2.487 (CI: 0.771-8.699; p = 0.154)]. After multivariate analysis, only mRS > 2 remained as significant independent predictors of poor functional outcome within 3 months amongst hemorrhagic stroke patients (p < 0.05) (Table 5).

**Table 5:** Independent predictors of poor outcome within 3 months post stroke

Variable	Functional Outcome		Univariate analysis		Multivariate analysis	
	Good	Poor	Unadjusted OR (95% CI)	P Value	Adjusted OR (95% CI)	P Value
AGE > 45 years	48(76.2)	34(82.9)	1.518(0.559-4.122)	0.411	--	--
Male gender	44(69.8)	23(56.1)	0.552(0.243-1.251)	0.153	0.799 (0.228-2.798)	0.726
Unemployed	40(63.5)	22(53.7)	0.666(0.299-1.482)	0.318	--	--
Unmarried	11(17.5)	6(14.6)	0.810(0.274-2.394)	0.703	--	--
Insurance	23(36.5)	10(24.4)	0.561(0.233-1.350)	0.194	0.858 (0.244-3.016)	0.811
Hypertension	62(98.4)	41(100.0)	Undefined	1.000	--	--
Diabetes Mellitus	13(20.6)	8(19.5)	0.932(0.348-2.495)	0.889	--	--
Smoking	15(23.8)	9(22.0)	0.900(0.352-2.303)	0.826	--	--
Alcohol abuse	36(57.1)	17(41.5)	0.531(0.239-1.179)	0.118	0.555 (0.195-1.583)	0.271
Dyslipidemia	46(73.0)	33(80.5)	1.525(0.589-3.950)	0.384	--	--
OBESITY	18(28.6)	12(29.3)	1.035(0.435-2.461)	0.939	--	--
Metabolic syndrome	17(27.0)	11(26.8)	0.992(0.409-2.409)	0.986	--	--
CAD	4(6.3)	0(0.0)	Undefined	0.152	--	--
Atrial fibrillation	2(3.2)	0(0.0)	Undefined	0.518	--	--
Heart disease	6(9.5)	0(0.0)	Undefined	0.042	--	--
Valvulopathy	3(4.8)	0(0.0)	Undefined	0.277	--	--
Recurrent stroke	8(12.7)	8(19.5)	1.667(0.571-4.863)	0.347	--	--
CKD	1(1.6)	0(0.0)	Undefined	1.000*	--	--
GCS<9	1(1.6)	5(12.2)	8.611(0.968-76.635)	0.034*	2.486 (0.208-29.705)	0.472
Hyperuricemia	15(23.8)	15(36.6)	1.846(0.781-4.364)	0.187	2.487 (0.711-8.699)	0.154
Complications	18(28.6)	19(46.3)	2.159(0.949-4.912)	0.064	1.823 (0.559-5.940)	0.319
NIHSS>14	12(19.0)	19(46.3)	3.671(1.524-8.838)	0.003	1.079 (0.348-3.348)	0.895
mRS>2	29(46.0)	40(97.0)	46.897(6.056-362.566)	<0.001	36.713 (4.280-314.931)	0.001
Hyperlycemia	15(23.8)	9(22.0)	1.934(0.678-5.511)	0.212	--	--
Elevated urea	8(12.7)	9(22.0)	1.934(0.678-5.511)	0.212	--	--
Elevated creatinine	19(30.2)	7(17.1)	0.478(0.180-1.265)	0.132	0.289 (0.068-1.233)	0.094
High LDLc	42(66.7)	34(82.9)	2.429(0.923-6.391)	0.068	1.028 (0.232-4.551)	0.971
High TC	24(38.1)	22(53.7)	1.882(0.848-4.175)	0.118	2.323 (0.642-8.402)	0.199
High TG	7(11.1)	7(17.1)	1.647(0.532-5.104)	0.384	--	--
Decreased HDLc	39(61.9)	22(53.7)	0.713(0.321-1.581)	0.404	--	--

CAD/IHD; coronary artery/ischemic heart disease, CKD; chronic kidney disease, GCS; Glasgow coma score, NIHSS: National Institute of Health Stroke Scale, mRS: modified Rankin scale, HDL: High density lipoprotein cholesterol, LDL: Low density lipoprotein cholesterol

## Discussion

According to our findings, we observed that the prevalence of hyperuricemia amongst hemorrhagic stroke patients was 34.4% (76/221). Secondly, SUA concentrations had positive significant correlations with SBP, DBP, creatinine, urea, triglycerides and LDL cholesterol while significant associations were observed between high SUA levels and the following CVRFs; alcohol abuse, dyslipidemia, obesity, metabolic syndrome and chronic kidney disease. Thirdly, we equally observed that hyperuricemia was not an independent predictor of stroke death and poor functional outcome amongst hemorrhagic stroke patients. Most epidemiological studies have reported a significant association between elevated SUA and increased cerebrovascular disease [22]. However, many studies have evaluated the prognostic significance of SUA in acute ischemic stroke and acute stroke (both ischemic and hemorrhagic stroke) but very few studies have reported on the impact of SUA on the prognosis of hemorrhagic stroke [15, 17, 23, 24]. In Ghana, Sarfo *et al.*. in an observational prospective study reported the prevalence of hyperuricemia of 46.3% amongst acute stroke patients and similar to our findings, early mortality was not significantly associated with hyperuricemia in hemorrhagic stroke [14]. Similarly in Iran, Mehrpour et al., reported the prevalence of 47.3% amongst 55 patients (46 ischemic and 9 hemorrhagic stroke patients) [25]. Conversely, the prevalence of hyperuricemia in patients with ischemic stroke is even higher as demonstrated by Koppula *et al.* [13]. With regards to the relationship between SUA and CVRFs, our findings are in accordance to the Iranian study where a significant negative correlation between SUA with the age of patients and hyperuricemia was associated increased amounts of TG and LDLc [25]. Furthermore our findings are also similar to those reported in a population-based cross-sectional study conducted by Qin *et al.*, in Shanghai, where SUA levels were positively associated with BMI, waist circumference, triglycerides and negatively associated with HDL-cholesterol [26]. Contrarily, no significant association was seen between these modifiable CVRFs and high SUA in stroke patients in the Ghanaian study [14]. This difference can be accounted for by differences in the methodology and the sample size in the Ghanaian study was small (147). Therefore, it is possible to conclude that this high prevalence of hyperuricemia may simply reflect its association or correlation with CVRFs present amongst hemorrhagic stroke patients.

In our study, we found no statistically significant correlation between SUA level and NIHSS and between SUA level and mRS in hemorrhagic stroke. We also noted a weak negative significant correlation between Glasgow coma score and SUA concentration. Similarly, Bandyopadhyay *et al.*. in India found a statistically insignificant weak positive correlation between uric acid level and change of Mathew score in hemorrhagic stroke patients [16]. Unlike our study, Ryu *et al.*, in Korea demonstrated that SUA is independently associated with the presence of cerebral microbleeds (CMBs) and the relation between uric acid and CMBs was robust in hypertensive patients [27]. Holme *et al.*. concluded that increasing SUA levels were associated with increased risk of both hemorrhagic and ischemic strokes [28]. Also, according to Bandyopadhyay et al., in New Delhi, amongst 96 acute stroke patients, high SUA level was insignificantly associated with poor neurological outcome in patients with hemorrhagic stroke [16]. In a Congolese cohort, hyperuricaemia was found to be predictor of stroke and all-cause mortality [29]. In 2009, Kim *et al.*, showed that patients with hyperuricemia were found to be at a significantly higher risk for both stroke incidence and mortality than controls with normal levels of SUA and after adjusting for known risk factors of stroke, the significance between hyperuricemia and stroke remained [22]. Strasak et al., in a prospective long-term study of 83 683 Austrian men demonstrated that increased SUA and the highest quintile is independently related to mortality from CHF and stroke [30]. It is worth noting that even though no significant association was found between uric acid and poor outcomes in hemorrhagic stroke, high uric acid levels in the acute phase of stroke could have occurred as a result its association with CVRFs such increasing age, hypertension, alcohol abuse, dyslipidemia, obesity and metabolic syndrome. Regardless of these findings, it is therefore possible to imply that hyperuricemia is a possible risk factor for stroke and primary prevention of stroke will be crucial in the reduction of mortality and morbidity related to hemorrhagic stroke amongst Cameroonians. The strength of this study was the fact that we prospectively explored different aspects of stroke outcomes in relation to uric acid amongst a cohort of patients with ICH but as a limitation, we were not able to explore certain variables that could have been more appropriate such the glycated hemoglobin (HbA1c). We used blood glucose levels in the study instead of the HbA1c hence overestimating the proportion of patients with diabetes mellitus as stress hyperglycemia could occur in acute conditions such as stroke. Despite these limitations, we have been able to assess the relationship between uric acid and stroke outcome.

## Conclusion

Our findings suggest that one third of patients present with hyperuricemia in the acute phase of hemorrhagic stroke. Hyperuricemia can act as a risk factor for stroke because of its relationship with CVRFs but it is not an independent predictor of the mortality and adverse outcome amongst black African hemorrhagic stroke patients.

### What is known about this topic

- The role of serum uric acid (SUA) in the acute phase of stroke remains controversial: there are studies which demonstrate their negative role in stroke outcome while others shows protective role;
- SUA is shown to influence the outcome of acute stroke in terms of mortality and functional outcome;
- Little is known in the black African leaving in Africa.

### What this study adds

- This study gives the prevalence of hyperuricemia among hemorrhagic stroke patients in a black African population;
- SUA does not influence the outcome of hemorrhagic stroke in terms of mortality and functional outcome;
- Even with increased level of SUA (quintile), there is no relation between SUA and hemorrhagic stroke outcome.
